# Saliva from nymph and adult females of *Haemaphysalis longicornis*: a proteomic study

**DOI:** 10.1186/s13071-015-0918-y

**Published:** 2015-06-24

**Authors:** Lucas Tirloni, Mohammad Saiful Islam, Tae Kwon Kim, Jolene K. Diedrich, John R. Yates, Antônio F. M. Pinto, Albert Mulenga, Myung-Jo You, Itabajara Da Silva Vaz

**Affiliations:** Centro de Biotecnologia, Universidade Federal do Rio Grande do Sul, Porto Alegre, RS Brazil; Department of Veterinary Parasitology, College of Veterinary Medicine and Bio-safety Research Centre, Chonbuk National University, Jeonju, Republic of Korea; Department of Medicine, Surgery and Obstetrics, Faculty of Veterinary and Animal Science, Hajee Mohammad Danesh Science and Technology University, Dinajpur, Bangladesh; Department of Chemical Physiology, The Scripps Research Institute, La Jolla, CA USA; Centro de Pesquisas em Biologia Molecular e Funcional, Instituto Nacional de Ciência e Tecnologia em Tuberculose (INCT-TB), Pontifícia Universidade Católica do Rio Grande do Sul (PUCRS), Porto Alegre, RS Brazil; Department of Veterinary Pathobiology, College of Veterinary Medicine, Texas A&M University, College Station, TX USA; Faculdade de Veterinária, Universidade Federal do Rio Grande do Sul, Porto Alegre, RS Brazil

**Keywords:** Tick, Proteomic, Saliva, Tick-host relationship

## Abstract

**Background:**

*Haemaphysalis longicornis* is a major vector of *Theileria* spp*.*, *Anaplasma phagocytophilum, Babesia* spp*.* and *Coxiella burnetti* in East Asian countries. All life stages of ixodid ticks have a destructive pool-feeding style in which they create a pool-feeding site by lacerating host tissue and secreting a variety of biologically active compounds that allows the tick to evade host responses, enabling the uptake of a blood meal. The identification and functional characterization of tick saliva proteins can be useful to elucidate the molecular mechanisms involved in tick development and to conceive new anti-tick control methods.

**Methods:**

*H. longicornis* tick saliva was collected from fully engorged nymphs and fully engorged adults induced by dopamine or pilocarpine, respectively. Saliva was digested with trypsin for LC-MS/MS sequencing and peptides were searched against tick and rabbit sequences.

**Results:**

A total of 275 proteins were identified, of which 135 were tick and 100 were rabbit proteins. Of the tick proteins, 30 proteins were identified exclusively in fully engorged nymph saliva, 74 in fully engorged adult females, and 31 were detected in both stages. The identified tick proteins include heme/iron metabolism-related proteins, oxidation/detoxification proteins, enzymes, proteinase inhibitors, tick-specific protein families, and cytoskeletal proteins. Proteins involved in signal transduction, transport and metabolism of carbohydrate, energy, nucleotide, amino acids and lipids were also detected. Of the rabbit proteins, 13 were present in nymph saliva, 48 in adult saliva, and 30 were present in both. The host proteins include immunoglobulins, complement system proteins, antimicrobial proteins, serum albumin, peroxiredoxin, serotransferrin, apolipoprotein, hemopexin, proteinase inhibitors, and hemoglobin/red blood cells-related products.

**Conclusions:**

This study allows the identification of *H. longicornis* saliva proteins. In spontaneously detached tick saliva various proteins were identified, although results obtained with saliva of fully engorged ticks need to be carefully interpreted. However, it is interesting to note that proteins identified in this study were also described in other tick saliva proteomes using partially engorged tick saliva, including hemelipoprotein, proteases, protease inhibitors, proteins related to structural functions, transporter activity, metabolic processes, and others. In conclusion, these data can provide a deeper understanding to the biology of *H. longicornis*.

**Electronic supplementary material:**

The online version of this article (doi:10.1186/s13071-015-0918-y) contains supplementary material, which is available to authorized users.

## Background

The hard tick *Haemaphysalis longicornis* is a medically and veterinary important vector of many tick-borne disease (TBD), transmitting pathogens such as *Ehrlichia chaffeensis* [1], *Anaplasma bovis* [2], *A. phagocytophilum* [3], *Coxiella burnetii* [4], and Spotted fever group rickettsiae [5]. Of significant veterinary importance, this tick species is considered the primary vector of theileriosis caused by *Theileria* spp. and of babesiosis caused by *Babesia* spp. in both sheep and cattle in East Asia [6, 7]. As a three-host tick, it has a wide range of hosts, from birds and lagomorphs (as immature ticks), and large domestic and wild mammals (as adult parasites). This tick is vastly distributed throughout Eastern Asian countries such as China, Korea, Japan, New Zealand, and Australia [8].

The tick feeding process is initiated when the tick engages and attaches onto its host. As a pool feeder, upon attachment the tick determines a suitable feeding site, and prepares it by lacerating small blood vessels. Feeding occurs by sucking up the blood that flows to the pool. This feeding style triggers host defense mechanisms such as pain or itching, hemostasis, inflammatory reactions, tissue repair, and immune rejection [9–12]. To control the feeding site and counteract the host defenses, ticks secrete and inject saliva into its host, of which contains hundreds of different proteins [7, 13–16] and other pharmacologically active molecules that confer anti-hemostatic, anti-inflammatory and immunomodulatory properties, supporting blood feeding [17–20].

During the feeding process, infected ticks may transmit TBD-causing pathogens. Besides being a critical component of the feeding process, saliva has also been shown to play a role in pathogen transmission [21]. Therefore, the identification and characterization of novel *H. longicornis* saliva proteins could point to candidates for the development of anti-tick and transmission-blocking vaccines [22–26] and of new pharmacological active molecules for medical application [18, 20, 27–29].

Currently the saliva proteome of *Amblyomma americanum*, *Ixodes scapularis*, *Ornithodoros moubata*, *Rhipicephalus sanguineus* sensu lato, *R. microplus*, and *Dermacentor andersoni* [13–16, 30–33] tick species have been analyzed. However, *H. longicornis* tick saliva proteome has not been the object of any analysis. The objectives of this study were to identify secreted proteins in the saliva of fully engorged nymphs (nymphs) and fully engorged adult females (adults) of *H. longicornis* ticks, comparing the protein profile of these developmental tick stages to evaluate the variation in tick saliva during feeding of different life stages. This affords to identify tick saliva proteins shared by the two developmental stages. Such proteins may play an important role in the success of both developmental stages in their feeding cycle. The novel catalog of tick saliva proteins identified in this study provides a deeper understanding to the biology of *H. longicornis*.

## Methods

### Ethics statement ethical approval

All animals used in these experiments were housed in Laboratory of Veterinary Parasitology, College of Veterinary Medicine and Bio-Safety Research Institute in Chonbuk National University, Jeonju 561–756, Republic of Korea. All animal studies and protocols are in agreement with the ethical principles for animal research and approved by the Chonbuk Animal Care and Use Committee (CBNU 2015–003).

### Ticks and saliva collection

The Jeju strain of the hard tick *H. longicornis* has been maintained on rabbits in our laboratory since 2003. To feed, *H. longicornis* ticks were placed onto the ears of specific pathogen-free (SPF) New Zealand White rabbits. Ticks were restricted to the ear using cloth pocket-like socks attached on ear ending with tape. Approximately 150 nymphs and 150 adults were placed in their respective feeding apparatuses and allowed to feed upon full engorgement and spontaneous detachment. Saliva was collected from 90 nymphs and 50 adults that were fully engorged and that detached from the rabbits spontaneously. Ticks were rinsed with sterile distilled water and induced to salivate by dorsal injection (posterior to fourth coxae in the region of epimeral and anal plates) of 5.0 to 7.0 μL 0.2 % dopamine or 1.5 to 3.0 μl 2 % pilocarpine (in 10 mM Tris-EDTA buffer) for nymphs and adults, respectively. Injections were applied using a micro-fine especially homemade glass needle. Then, ticks were maintained at 37 °C in an 85 % relative humidity chamber, and saliva was periodically collected for approximately 4 h using a pipette tip. Tick saliva was lyophilized and stored at −80 °C for LC-MS/MS analysis. Saliva protein concentrations were determined by Qubitfluorometer (Life Technologies, Carlsbad, CA, USA).

### Protein digestion and sample preparation

Saliva of *H. longicornis* nymphs and adult females was digested in solution with trypsin. Lyophilized salivary proteins were dissolved in 8 M urea/0.1 M Tris, pH 8.5, reduced with 5 mMTris(2-carboxyethyl)phosphine hydrochloride (TCEP, Sigma-Aldrich, St Louis, MO, USA) and alkylated with 25 mM iodoaceamide (Sigma-Aldrich). Proteins were digested overnight at 37 °C in 2 M urea/0.1 M Tris pH 8.5, 1 mM CaCl_2_ with trypsin (Promega, Madison, WI, USA) at a final 1:20 ratio (enzyme:substrate). Digestions were stopped with formic acid (5 % final concentration) and centrifuged for debris removal.

### Precolumns and analytical columns

Reversed phase pre-columns were prepared by first creating a Kasil frit at one end of a deactivated 250-μm ID/360-μm OD capillary (Agilent Technologies, Santa Clara, CA, USA). Kasil frits were prepared by dipping 20 cm capillary in 300 μL Kasil 1624 (PQ Corporation, Malvern, PA, USA) and 100 μL formamide solution, curing at 100 °C for 3 h, and cutting the frit to a length of 2 mm. Pre-columns were packed in-house with 5 μm ODS-AQ C18 (YMC America, INC., Allentown, PA, USA) particles from particle slurries in methanol upon reaching the height of 2 cm. Analytical reversed phase columns were assembled by pulling a 100-μm ID/360-μm OD (Molex Polymicro Technologies™, Austin, TX, USA) silica capillary to a 5 μm ID tip. The same packing material was packed directly into the pulled column until the length of 20 cm was reached. Reversed phase precolumns and analytical columns were connected using a zero-dead volume union (IDEX Corp., Upchurch Scientific, Oak Harbor, WA, USA).

### LC-MS/MS

Peptide mixtures were analyzed by nanoflow liquid chromatography mass spectrometry using an Easy NanoLC II and a Q Exactive mass spectrometer (Thermo Scientific, Waltham, MA, USA). Peptides eluted from the analytical column were electrosprayed directly into the mass spectrometer. Buffer A and B consisted of 5 % acetonitrile/0.1 % formic acid and 80 % acetonitrile/0.1 % formic acid, respectively. The flow rate was set to 400 nL/min. *H. longicornis* digested saliva samples (1.5 μg per injection) were separated in 155 min chromatographic runs, with linear gradient from 1 to 10 % of buffer B for 10 min followed by an increase to 40 % of buffer B in 100 min, an increase to 50 % of buffer B in 10 min and finally an increase to 90 % of buffer B for additional 10 min. Column was held at 90 % of buffer B for 10 min, reduced to 1 % of buffer B and re-equilibrated prior to the next injection.

The mass spectrometer was operated in a data dependent mode, collecting a full MS scan from 400 to 1200 m/z at 70,000 resolution and an AGC target of 1 × 10^6^. The 10 most abundant ions per scan were selected for MS/MS at 17,500 resolution and AGC target of 2 × 10^5^ and an underfill ratio of 0.1 %. Maximum fill times were 20 and 120 ms for MS and MS/MS scans, respectively, with dynamic exclusion of 15 s. Normalized collision energy was set to 25.

### Data analysis

Tandem mass spectra were extracted from raw files using RawExtract 1.9.9.2 [34] and searched with ProLuCID [35] against a combined non-redundant database containing (i) Ixodidae database from NCBI; (ii) *R. microplus* transcriptome database (Rm-INCT-EM, containing 22,009 sequences produced by our research group using Illumina Sequencing technology – BioProject ID PRJNA232001 at Transcriptome Shotgun Assembly (TSA) database – GenBank); (iii) *Oryctolagus cuniculus* database from SwissProt and (iv) reverse sequences of all database entries. Searches were done using an Integrated Proteomics Pipeline – IP2 (Integrated Proteomics Applications) for The Scripps Institute (La Jolla, CA, USA). The search space included all fully-tryptic and half-tryptic peptide candidates. Carbamidomethylation on cysteine was used as static modification. Data was searched with 50-ppm precursor ion tolerance and 20-ppm fragment ion tolerance.

The validity of the peptide spectrum matches (PSMs) generated by ProLuCID was assessed using Search Engine Processor (SEPro) [36]. Identifications were grouped by charge state and tryptic status, resulting in four distinct subgroups. For each group, ProLuCIDXCorr, DeltaCN, DeltaMass, ZScore, number of peaks matched and Spec Count Score values were used to generate a Bayesian discriminator. The identifications were sorted in a nondecreasing order according to the discriminator score. A cutoff score was established to accept a false discovery rate (FDR) of 1 % based on the number of decoys. This procedure was independently performed on each data subset, resulting in a false-positive rate that was independent of tryptic status or charge state. Additionally, a minimum sequence length of six residues per peptide was required. Results were post-processed to only accept PSMs with < 10 ppm precursor mass error.

A Volcano plot was generated by a pairwise comparison between nymphs and fully engorged female *H. longicornis* tick saliva using the TFold module from PatternLab for Proteomics platform [37]. The following parameters were used to select differentially expressed proteins: proteins were grouped by maximum parsimony, spectral count data was normalized using normalized spectral abundance factor (NSAF) [38], and two nonzero replicate values were required for each condition (at least two out of four replicates). A BH q-value was set at 0.02 (2 % FDR). A variable fold-change cutoff for each individual protein was calculated according to the *t*-test *p*-value using an F-Stringency value automatically optimized by the TFold software. Low abundant proteins were removed using an L-stringency value of 0.4.

Venn diagrams were manually generated from the output of PatternLab’s Birds Eye view report. Proteins were grouped by maximum parsimony and the presence of proteins in at least two out of four replicates was required for each condition.

### Functional annotation and classification

To gain insight on the nature of the identified protein sequences, BLASTp searches against several databases were performed. To check the identity of tick saliva proteins detected, several databases were used for screening: non-redundant (NR), Acari and refseq-invertebrate from NCBI; Acari from Swissprot; the GeneOntology (GO) FASTA subset [39]; MEROPS database; and the conserved domains database of NCBI [40] containing the KOG [41], PFAM [42], and SMART motifs [43]. To check rabbit proteins identity, we used *Oryctolagus cuniculus* and refseq-vertebrates databases from NCBI; the conserved domains database of NCBI [40] containing the KOG [41], PFAM [42], and SMART motifs [43]; and the GeneOntology (GO) FASTA subset [39]. To functionally classify the protein sequences, a program written and provided by Dr. José M. C. Ribeiro in Visual Basic 6.0 (Microsoft, Redmond, Washington, USA) was used [44]. The functionally annotated catalog for each dataset was manually curated and plotted in a hyperlinked Excel spreadsheet (Additional file 1: Table S1 and Additional file 2: Table S2).

## Results and discussion

*H. longicornis* is the primary tick known to transmit disease pathogens to humans and animals in East Asian countries [45]. Like several other blood-sucking parasites, its saliva secretion is a mixture of proteins produced in different salivary gland acinar cells [46]. Tick salivation can be studied *in vivo* by injection of dopamine, a neurotransmitter that stimulates fluid secretion by salivary gland, or by using pilocarpine, a cholinomimetic agent that induces the release of dopamine from the salivary nerves, resulting in salivation [47–49]. In *Ornithodoros moubata*, comparative analysis by SDS-PAGE of several pilocarpine- and dopamine-induced saliva batches demonstrated reproducibility between both protocols used to induce tick salivation [13]. We have tested the use of pilocarpine and/or dopamine in both adult and nymph saliva collection. However, using pilocarpine for nymph saliva collection did not result in a substantial amount of saliva. When dopamine was used in adult ticks, the saliva acquired a darker color (data not shown). This observation using dopamine for adult saliva stimulation is in accordance with results described for *R. sanguineus* s.l. [15]. However, differently from data obtained for *R. sanguineus* s.l. adult tick saliva, we successfully collected and identified proteins from *H. longicornis* nymph saliva induced by dopamine. These differences could be associated to stage and/or species of ticks used in the salivation induction. Few tick saliva proteomes have been published, most of which using adult ticks and, therefore, knowledge about nymph saliva collection is not available.

At the time study was carried out, *H. longicornis* genome and transcriptome sequences were not available, therefore we screened against available Ixodidae databases from NCBI and an in-house *R. microplus* transcriptome database to identify tick salivary proteins by shotgun proteomics. Additionally, saliva was screened for host proteins using available rabbit protein sequences from Uniprot. The comparison in proteomic content between samples from different life cycle stages may show unique or increased levels of particular proteins that are important for tick biology. Moreover, it is possible to provide information about semi-quantitative variations on the levels of the specific proteins. Comparative proteomics of spontaneously detached fully engorged nymph and fully engorged adult females *H. longicornis* have shown alterations in protein salivary content through different life cycle stages, generating new insights into tick physiology. The main objective in this study was to evaluate the *H. longicornis* saliva proteome, showing saliva protein content in nymphs, as an immature stage, and in fully engorged adult females, as an experimental end-point, evaluating not only differences, but also the similarities in saliva contents at different stages of tick development. It is important to observe that analyses of saliva of fully engorged ticks have to be carefully interpreted, since they are at the end of the feeding process. However, this analysis can provide useful information, since various categories of proteins identified in this study were also described in other tick saliva proteomes using partially engorged tick saliva [13–16], including: proteins related to heme/iron metabolism (hemelipoprotein, ferritin), proteases (cathepsin, trypsin-like, metalloprotease), protease inhibitors (serpin, cystatin, alpha-2-macroglobulin, TIL), proteins related to structural functions, transporter activity, metabolic processes, protein modification machinery (heat shock proteins), and others. In addition, other proteins from fully engorged ticks have been characterized as anticoagulant molecules, like microphilin [27], BmAP [28], and haemalin [29]. Therefore, despite the limitation in using fully engorged adult saliva, the biological interpretation of these data can provide a deeper understanding to the biology of *H. longicornis*.

### An overview of identified saliva proteins

**Fig. 1:**
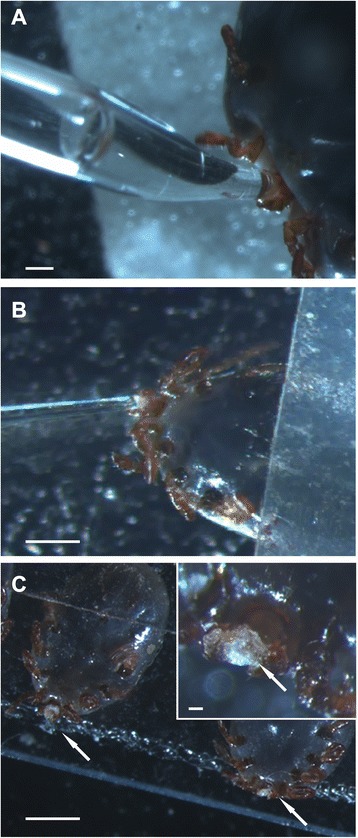
*Haemaphysalis longicornis* saliva collection. **a** Saliva collection from a fully engorged adult female. **b** Saliva collection from a fully engorged nymph. **c** After application of dopamine, saliva appears as crystals in nymph mouthparts (arrows). Insert shows a magnified image of crystals in mouthparts. Bars = 100 μm

**Fig. 2:**
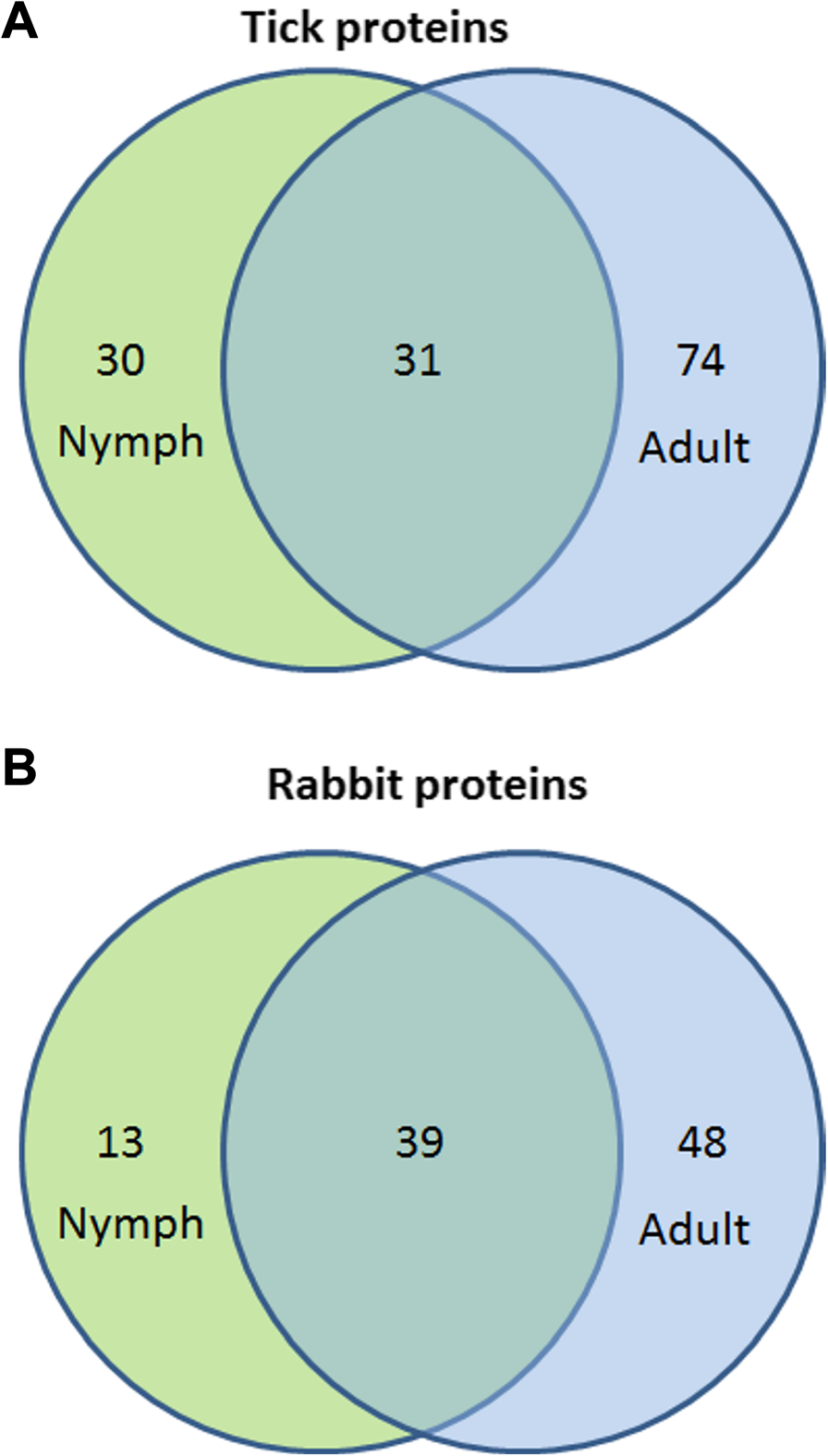
Venn diagram of *Haemaphysalis longicornis* saliva proteins identified in fully engorged nymphs (nymph) and fully engorged adult females. **a** Tick and **b** rabbit proteins identified in tick saliva. The overlap between circles shows the proteins present in both stages

**Fig. 3:**
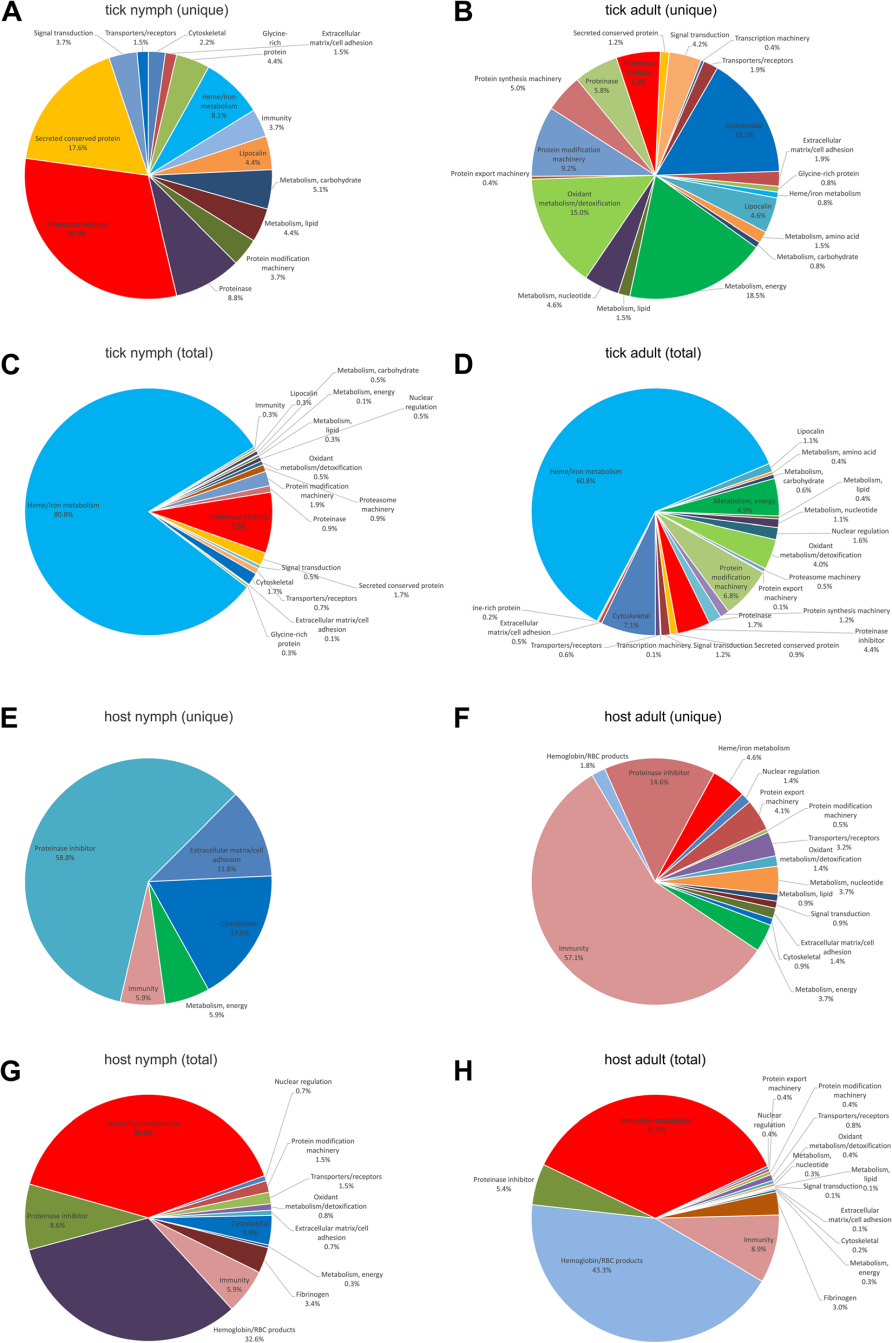
Functional classification of *Haemaphysalis longicornis* salivary proteins identified in fully engorged nymph (nymph) and fully engorged adult females (adult). **a** Tick proteins detected in nymph (**a** and **c**) and adult (**b** and **d**). Proteins were divided as detected both in nymph (**c**) and adult (**d**) and proteins detected exclusively in nymph (**a**) or adult (**b**) and classified in groups according to their function and/or protein family. Pie charts represent the percentage of proteins found in each group with respect to normalized spectral counting for each sample. **b** Host proteins detected in nymph (**e** and **g**) and adult (**f** and **h**). Proteins were divided as detected both in nymph (**g**) and adult (**h**) and proteins detected exclusively in nymph (**e**) or adult (**f**) and classified in groups according to their function and/or protein family. Pie charts represent the percentage of proteins found in each group with respect to normalized spectral counting for each sample. Keratin was removed before data interpretation. (Additional file 3: Figure S1 shows data with keratin)

**Fig. 4:**
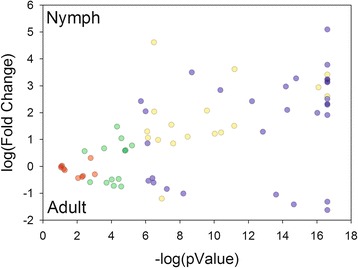
Volcano plot abundance changes analysis of *Haemaphysalis longicornis* saliva proteins identified both in fully engorged nymph (nymph) and fully engorged adult females (adult). Each point represents the difference in expression (log2 fold difference) between nymph and adult plotted against the level of statistical significance. Proteins represented by (blue dot) had an identification that satisfied both fold and statistical criteria; (yellow dot) had an identifications that was filtered out by the L-stringency; (green dot) had an identification satisfied the fold criteria but, most likely, this happened by chance; and (red dot) had identification did not meet the fold and *p*-value criteria

Tick saliva was obtained by dopamine and pilocarpine stimulations from *H. longicornis* nymphs and adult females, respectively. The saliva accumulated in the mouthparts was periodically collected from the ticks using a pipette tip (Fig. 1a and b). Crystal particles formed around the nymph mouthpart were also collected (Fig. 1c). Collected saliva was subjected to tryptic digestion and analyzed by shotgun proteomics in quadruplicate. A total of 135 proteins were identified matching tick databases, and 100 proteins matching rabbit database (Fig. 2 and Tables 1, 2 and 3). The identified tick proteins were classified and divided into groups according to their putative functions (Fig. 3 and Tables 1, 2 and 3), consistent with previously published tick sialomes [9]. In the set of tick specific proteins, 30 proteins were identified exclusively in nymph saliva, 74 proteins were identified exclusively in adult saliva, and 31 proteins were detected in both stages (Fig. 2). Of those 31 identified tick proteins detected in both stages, 11 had statistical differential expression confirmed (Fig. 4 and Table 4). This finding is discussed below. These proteins identified in nymph saliva as well as in adult saliva can be secreted through developmental stages throughout the tick feeding process, since proteomic studies using saliva from other tick species collected during feeding process have described the presence of similar classes of proteins as identified in this study [14–16]. Moreover, some of these proteins have been used in anti-tick vaccination experiments, e.g. glutathione S-transferase [50, 51], cystatin [52], ferritin [53, 54], serpins [23, 55–59], and hemelipoproteins [60, 61].

**Table 1:** Tick and host proteins identified exclusively in nymph saliva

Contig number^a^	Annotation	Class	Spec count
TICK			
BAF36722	glycine-rich cell wall structural protein	Glycine-rich protein	1.00
Rm-6837	glycine proline-rich secreted protein	Glycine-rich protein	5.25
Rm-21891	actin-depolymerizing factor	Cytoskeletal	3.00
JAA63693	substrate adhesion-dependent cell spreading	Extracellular matrix/cell adhesion	2.50
JAA55046	toll-like receptor 5	Immunity	5.00
ACX33152	catalytically inactive chitinase-like lectins	Metabolism, carbohydrate	5.00
JAA63210	lysosomal alpha-mannosidase-like isoform *X*2	Metabolism, carbohydrate	2.00
JAA54599	phospholipase A2	Metabolism, lipid	6.00
Rm-32843	lysosomal & prostatic acid phosphatase	Protein modification machinery	4.66
BAF31119	secreted protein	Secreted conserved protein	7.75
AEO33293	antigen 5/SCP	Secreted conserved protein	5.75
AEO36255	secreted protein	Secreted conserved protein	1.66
JAA60619	secreted protein	Secreted conserved protein	5.50
Rm-9069	secreted protein	Secreted conserved protein	1.50
BAE02551	lipocalin	Lipocalin	5.50
BAF43801	longipain	Proteinase	5.75
DAA34687	cathepsin L-like	Proteinase	1.50
JAA62284	tick serine protease	Proteinase	1.50
Rm-44814	serine carboxypeptidase	Proteinase	2.00
ACJ26770	alpha-2-macroglobulin	Proteinase inhibitor	27.75
EEC05896	serpin	Proteinase inhibitor	1.00
AEO34218	serpin	Proteinase inhibitor	2.33
AEO34349	serpin	Proteinase inhibitor	4.75
JAB70612	alpha-2-macroglobulin	Proteinase inhibitor	1.25
Rm-12491	serpin	Proteinase inhibitor	4.00
AEO35364	cystatin	Proteinase inhibitor	1.25
EEC02492	TGF-beta-induced protein ig-h3	Signal transduction	5.25
JAA58175	chorion peroxidase	Heme/iron metabolism	5.50
BAN13552	ferritin	Heme/iron metabolism	5.00
JAA54618	metabotropic glutamate receptor	Transporters/receptors	2.25
RABBIT			
G1T4V7	myosin tail	Cytoskeletal	6.25
G1T4A5	collagen alpha-1 (I) chain-like	Extracellular matrix/cell adhesion	4.00
P25227	alpha-1-acid glycoprotein 1	Immunity	1.66
G1TJG6	glyceraldehyde-3-phosphate dehydrogenase	Metabolism, energy	1.50
G1TFX2	alpha-1-antiproteinase	Proteinase inhibitor	10.00
Q28665	alpha-1-antiproteinase	Proteinase inhibitor	10.00
G1SDN2	keratin 17	Keratin	35.50
G1SHY2	keratin 75 - extracellular vesicular exosome	Keratin	71.00
G1SHZ4	keratin type II cytoskeletal 7	Keratin	48.50
G1SPP3	keratin type II cytoskeletal 4	Keratin	59.50
G1SUH1	keratin type II cytoskeletal 72 isoform X1	Keratin	7.66
G1T8T1	keratin type I cytoskeletal 28	Keratin	13.25
G1U758	keratin type I cytoskeletal 18	Keratin	16.25

^a^Protein and spectral count of host and tick proteins identified in nymph *Haemaphysalis longicornis* saliva. Annotation and accession numbers of best match identities obtained using BLASTP against several protein databases can be checked with more details in Additional file 1: Table S1 and Additional file 2: Table S2

**Table 2:** Tick and host proteins identified *exclusively* in adult saliva

Contig number^a^	Annotation	Class	Spec count
TICK			
JAA54320	glycine-rich protein	Glycine-rich protein	1.50
EEC14126	radixin/ezrin/moesin	Cytoskeletal	2.00
ACX53929	putative beta thymosin	Cytoskeletal	1.33
DAA34555	microtubule-binding protein	Cytoskeletal	1.00
AEO32824	actin depolymerizing factor	Cytoskeletal	1.50
AEO33976	beta tubulin	Cytoskeletal	6.50
AFR32950	paramyosin	Cytoskeletal	5.33
JAB76162	dynein light chain	Cytoskeletal	1.00
JAB80373	myosin class i heavy chain	Cytoskeletal	12.25
AAY42205	troponin T	Cytoskeletal	1.00
Rm-1533	beta tubulin partial	Cytoskeletal	6.33
Rm-80704	myosin class ii heavy chain	Cytoskeletal	4.67
ACF35539	glutathione S-transferase	Oxidant metabolism/detoxification	6.50
ACG76272	aldehyde dehydrogenase	Oxidant metabolism/detoxification	1.00
AAQ74441	glutathione S-transferase	Oxidant metabolism/detoxification	1.50
AEO33057	sulfotransferase	Oxidant metabolism/detoxification	2.00
AEO35358	catalase	Oxidant metabolism/detoxification	8.00
JAA63098	putative aldehyde dehydrogenase	Oxidant metabolism/detoxification	3.00
Rm-14504	aldehyde dehydrogenase	Oxidant metabolism/detoxification	14.75
Rm-46289	aldehyde dehydrogenase	Oxidant metabolism/detoxification	2.00
ADN34303	cuticular protein	Extracellular matrix/cell adhesion	4.75
AEO34631	phosphoserine aminotransferase	Metabolism, amino acid	1.50
Rm-73466	homocysteine S-methyltransferase	Metabolism, amino acid	2.50
AEO32930	transketolase	Metabolism, carbohydrate	2.33
ACH88101	glyceraldehyde-3-phosphate dehydrogenase	Metabolism, energy	2.67
AEO32901	ATP synthase subunit beta	Metabolism, energy	7.75
AEO34579	3-phosphoglycerate dehydrogenase	Metabolism, energy	3.75
AEO35473	Isocitrate dehydrogenase NADP	Metabolism, energy	6.00
JAA60091	3-phosphoglycerate dehydrogenase	Metabolism, energy	3.33
JAA60302	Isocitrate dehydrogenase NADP	Metabolism, energy	7.00
JAA62224	imp cyclohydrolase/methylglyoxal synthase	Metabolism, energy	8.75
JAA66712	C-1-tetrahydrofolate synthase	Metabolism, energy	2.00
JAA68969	glyceraldehyde-3-phosphate dehydrogenase	Metabolism, energy	2.50
JAB74970	cytochrome B5	Metabolism, energy	1.33
Rm-26196	Malate dehydrogenase	Metabolism, energy	2.67
Rm-18758	15-hydroxyprostaglandin dehydrogenase	Metabolism, lipid	1.50
Rm-73635	farnesoic acid o-methyltransferase	Metabolism, lipid	1.67
JAA59537	3’(2’)5’-bisphosphate nucleotidase	Metabolism, nucleotide	1.00
JAA60240	GDP dissociation inhibitor	Metabolism, nucleotide	1.50
	Inosine-5’-monophosphate dehydrogenase	Metabolism, nucleotide	4.75
Rm-32846	purine nucleoside phosphorylase transferase	Metabolism, nucleotide	2.00
Rm-6587	SAICAR synthase	Metabolism, nucleotide	2.00
AEO35504	annexin	Protein export machinery	1.00
BAF63673	protein disulfide-isomerase	Protein modification machinery	2.50
EEC14106	multifunctional chaperone	Protein modification machinery	2.50
JAA60036	heat shock protein	Protein modification machinery	1.50
JAA67522	heat shock protein hsp 90-alpha isoform	Protein modification machinery	2.50
JAB73342	methyltransferase	Protein modification machinery	1.50
JAB75807	cysteine s-methyltransferase	Protein modification machinery	2.00
Rm-24571	heat shock protein 70 cognate	Protein modification machinery	6.50
Rm-62470	heat shock-related protein	Protein modification machinery	3.75
JAA62377	gmp synthase	Protein modification machinery	2.50
AEO32591	elongation factor 1-alpha	Protein synthesis machinery	8.33
AEO32633	AAA+ ATPase	Protein synthesis machinery	1.00
Rm-50382	elongation factor 2-like	Protein synthesis machinery	4.00
AEO34773	secreted protein	Secreted conserved protein	3.00
AEO32884	lipocalin	Lipocalin	7.25
Rm-26539	lipocalin	Lipocalin	5.00
BAF51711	tick legumain	Proteinase	8.50
BAH86062	cathepsin L-like	Proteinase	2.33
JAA60171	metalloexopeptidase	Proteinase	3.00
JAA65860	CNDP dipeptidase	Proteinase	2.33
EEC20000	alpha-2-macroglobulin	Proteinase inhibitor	6.00
BAH02683	haemalin	Proteinase inhibitor	4.50
AEO35673	TIL	Proteinase inhibitor	5.00
BAG41813	cyclophilin 1	Signal transduction	3.50
AEO32842	regulatory protein mlp	Signal transduction	2.00
JAB75372	calmodulin	Signal transduction	1.67
Rm-69447	neuropeptide-like protein 31	Signal transduction	1.00
Rm-79606	Regucalcin	Signal transduction	2.50
Rm-154439	hemelipoprotein	Heme/iron metabolism	1.67
JAA62029	zinc finger protein	Transcription machinery	1.00
EEC05877	apolipophorin	Transporters/receptors	2.75
JAB78095	glutamate receptor 1-like	Transporters/receptors	1.67
RABBIT			
G1U9R8	gelsolin	Cytoskeletal	2.50
P27170	serum paraoxonase/arylesterase 1	Oxidant metabolism/detoxification	1.00
G1SR65	myeloperoxidase	Oxidant metabolism/detoxification	1.50
G1T049	collagen alpha-6(IV) chain	Extracellular matrix/cell adhesion	1.00
G1TZA1	type X collagen alpha 1-like	Extracellular matrix/cell adhesion	2.25
O77791	protein S100-A12	Immunity	4.50
P01840	Ig kappa-b4 chain C region	Immunity	10.25
P01870	Ig gamma-1 chain C region	Immunity	48.50
P04221	Ig mu chain C region	Immunity	4.00
G1SS91	complement C4-A	Immunity	3.75
G1T3X1	complement component C9	Immunity	2.00
G1TEC1	Ig kappa chain V-I	Immunity	4.75
G1TFU1	Ig kappa chain V-I	Immunity	5.25
G1TKP3	immunoglobulin lambda-like	Immunity	4.00
G1TM51	Ig kappa chain V-I	Immunity	4.00
G1TPF2	T-cell surface glycoprotein CD8 -like	Immunity	5.00
G1TPZ1	galectin-1	Immunity	2.33
G1TRW8	neutrophil gelatinase-associated lipocalin	Immunity	8.50
G1TUX5	C1q and TNF related protein 7-like	Immunity	2.50
G1TVN7	Ig kappa chain V-I	Immunity	5.75
G1TVZ5	T-cell surface glycoprotein CD8 -like	Immunity	1.50
Q9GK63	mammaglobin-B	Immunity	2.00
Q9GK67	secretoglobin	Immunity	3.67
U3KM01	Ig kappa chain V-I	Immunity	4.00
G1SQA8	ATP synthase mitochondrial precursor	Metabolism, energy	3.50
G1TS29	triosephosphate isomerase	Metabolism, energy	1.33
G1TYA7	L-lactate dehydrogenase B chain	Metabolism, energy	2.67
G1TB24	peroxysomal Fatty Acyl CoA Transporter	Metabolism, lipid	1.50
G1SN21	purine nucleoside phosphorylase	Metabolism, nucleotide	2.00
G1TXI1	nucleophosmin	Metabolism, nucleotide	3.33
G1U9T4	nucleoside diphosphate kinase B	Metabolism, nucleotide	3.33
G1U155	histone H2B type 1-like	Nuclear regulation	3.00
P51662	annexin A1	Protein export machinery	5.50
G1TDI4	transthyretin	Protein export machinery	3.00
G1SJZ9	thioredoxin	Protein modification machinery	1.33
G1SEK8	metalloendopeptidase inhibitor	Proteinase inhibitor	6.75
G1SIK0	antithrombin III	Proteinase inhibitor	8.67
G1SZA4	inter-alpha-trypsin inhibitor heavy chain H1	Proteinase inhibitor	2.00
G1TM88	alpha-1-antitrypsin	Proteinase inhibitor	2.33
G1U6R8	negative regulation of endopeptidase	Proteinase inhibitor	8.25
Q45GR2	alpha-2-antiplasmin	Proteinase inhibitor	3.50
G1SNP6	serine/threonine-protein kinase	Signal transduction	1.50
P09809	apolipoprotein A-I	Heme/iron metabolism	4.25
G1TFW8	lactotransferrin	Heme/iron metabolism	5.75
G1SU82	vitamin D-binding protein	Transporters/receptors	4.75
G1T4K1	sodium channel protein type 3 isoform X1	Transporters/receptors	2.00
G1TP66	keratin type II cuticular Hb3	Keratin	1.50
P00919	carbonic anhydrase 2	Hemoglobin/RBC products	3.50

^a^Protein and spectral count of host and tick proteins identified in adult *Haemaphysalis longicornis* saliva. Annotation and accession numbers of best match identities obtained using BLASTP against the several protein databases can be checked with more details in Additional file 1: Table S1 and Additional file 2: Table S2

**Table 3:** Tick and host proteins identified in nymph and adult saliva

Contig number	Annotation	Class	Spec count	
			Nymph	Adult
TICK				
ABQ96858	tropomyosin	Cytoskeletal	3.00	5.25
BAF98180	actin	Cytoskeletal	21.75	18.75
AEO32669	alpha tubulin	Cytoskeletal	3.25	11.00
AGC13075	glutathione peroxidase	Oxidant metabolism/detoxification	10.50	4.33
AEO34612	enolase	Metabolism, carbohydrate	2.00	4.25
Rm-10851	ATP synthase subunit alpha	Metabolism, energy	1.50	4.67
AEO34838	histone H4	Nuclear regulation	5.75	7.33
AEO34879	histone H2A	Nuclear regulation	4.00	10.00
AEO32095	heat shock 70 kDa protein	Protein modification machinery	5.50	5.67
AEO32791	heat shock protein	Protein modification machinery	9.75	11.00
AEO34048	protein disulfide-isomerase A6-like	Protein modification machinery	1.50	1.00
JAA62581	heat shock protein	Protein modification machinery	3.00	3.50
ADG86641	lysosomal acid phosphatase	Protein modification machinery	9.50	26.75
JAA73257	ubiquitin/40s ribosomal protein s27a	Proteasome machinery	17.25	4.75
ADK47399	secreted protein	Secreted conserved protein	2.67	3.00
AGH08176	AV422	Secreted conserved protein	4.75	4.25
BAE53722	aspartic protease	Proteinase	4.00	3.25
BAD11156|	serpin	Proteinase inhibitor	15.50	3.50
JAA60430	alpha-2-macroglobulin	Proteinase inhibitor	41.00	12.33
JAA64973	alpha-2-acroglobulin	Proteinase inhibitor	17.50	3.50
Rm-7619	alpha-2-macroglobulin	Proteinase inhibitor	26.25	9.00
ABZ89554.	cystatin	Proteinase inhibitor	6.33	2.67
Rm-69112	14–3–3 zeta	Signal transduction	4.33	2.33
BAG12081	hemelipoprotein	Heme/iron metabolism	796.25	323.25
BAJ21514	hemelipoprotein	Heme/iron metabolism	405.00	224.00
BAJ21515	hemelipoprotein	Heme/iron metabolism	98.50	6.25
BAL42280	hemelipoprotein	Heme/iron metabolism	141.75	60.75
JAA59652	hemelipoprotein	Heme/iron metabolism	19.67	14.25
ABD83654	hemelipoprotein	Heme/iron metabolism	12.50	12.25
Rm-72548	hemelipoprotein	Heme/iron metabolism	32.25	14.75
JAA61676	plexins functional semaphorin receptor	Transporters/receptors	12.50	2.50
RABBIT				
G1T229	filaggrin-2	Cytoskeletal	17.00	1.50
G1T6W7	catalase	Oxidant metabolism/detoxification	5.25	7.50
P16973	lysozyme C	Immunity	3.00	7.50
P25230	antimicrobial protein CAP18	Immunity	1.50	5.33
P50117	protein S100-A9	Immunity	3.75	3.00
G1SUZ1	complement C3	Immunity	5.25	15.00
G1SYM4	alpha-1B-glycoprotein	Immunity	3.75	3.00
G1THZ6	Ig gamma-1 chain C region	Immunity	15.00	62.00
G1T0Z2	histone H2A type 1-A	Nuclear regulation	4.00	5.67
G1T9M9	heat shock cognate 71 kDa protein	Protein modification machinery	9.00	10.00
G1SQ70	alpha-2-macroglobulin	Proteinase inhibitor	10.75	32.50
G1TFV7	alpha-1-antiproteinase	Proteinase inhibitor	10.25	37.25
Q07298	alpha-1-antiproteinase	Proteinase inhibitor	10.00	32.67
P19134	serotransferrin	Heme/iron metabolism	33.75	95.25
P20058	hemopexin	Heme/iron metabolism	12.00	36.50
G1SQ02	peroxiredoxin-1	Heme/iron metabolism	3.00	3.25
G1SWF6	haptoglobin - hemoglobin binding	Heme/iron metabolism	7.50	20.00
G1TVS4	hemopexin	Heme/iron metabolism	12.00	38.25
G1U9S2	serum albumin	Heme/iron metabolism	156.00	665.50
G1SGQ5	alpha-2-HS-glycoprotein	Transporters/receptors	3.00	1.50
G1SLY0	HCO3- transporter family	Transporters/receptors	1.50	3.00
U3KMC6	ceruloplasmin	Transporters/receptors	3.67	8.50
G1SKE3	keratin type II cytoskeletal 6A	Keratin	84.50	20.00
G1SUH8	keratin 2	Keratin	228.75	52.00
G1SWB8	keratin type I cytoskeletal 27	Keratin	23.67	14.00
G1SY72	keratin type II cytoskeletal	Keratin	68.50	17.33
G1T1V0	keratin type I cytoskeletal 10	Keratin	108.50	58.50
G1T1Y7	keratin type I cytoskeletal 14 isoform X1	Keratin	56.00	12.50
G1T4R6	keratin type I cytoskeletal 16	Keratin	57.50	13.25
G1T4S1	keratin 15	Keratin	51.75	17.00
G1TDN6	keratin type II cytoskeletal 5	Keratin	99.75	14.75
G1U754	histidine-rich glycoprotein	Heme/iron metabolism	11.75	25.25
G1U9I8	keratin type II cytoskeletal 1	Keratin	104.25	39.75
G1T0W8	fibrinogen beta chain	Fibrinogen	5.25	26.50
G1T0X2	fibrinogen alpha chain	Fibrinogen	9.00	13.50
G1TKX3	fibrinogen gamma chain	Fibrinogen	6.25	34.75
P01948	hemoglobin subunit alpha-1/2	Hemoglobin/RBC products	87.00	544.00
P02057	hemoglobin subunit beta	Hemoglobin/RBC products	100.75	517.50
P07452	carbonic anhydrase 1	Hemoglobin/RBC products	5.00	11.00

^a^Protein and spectral count of host and tick proteins identified both in nymph and adult *Haemaphysalis longicornis* saliva. Annotation and accession numbers of best match identities obtained using BLASTP against several protein databases can be checked with more details in Additional file 1: Table S1 and Additional file 2: Table S2

**Table 4:** Differential abundance of nymph and adult saliva proteins (determined using the TFold)

Annotation	Contig number^a^	Class	Fold change^b^	*p* value
TICK				

RABBIT				

TICK				

RABBIT				

TICK				

RABBIT				

TICK				

RABBIT				


Blue: identifications that satisfied both fold and statistical criteria

Yellow: identifications were filtered out by the L‐stringency

Green: identifications satisfied the fold criteria but, most likely, this happened by chance

Red: identifications did not meet the fold and *p*‐value criteria

^a^Accession numbers of best matches identities obtained using BLASTP against the non‐redundant protein database in GenBank

^b^Positive number means the fold increased expression in nymph (relation between nymph and adult). Negative number means the fold increased expression in adults (relation between adult and nymph)

The identified tick proteins include (i) heme/iron metabolism-related proteins, including hemelipoproteins and ferritin; (ii) proteins related to oxidation/detoxification functions, including glutathione S-transferase, aldehyde dehydrogenase, glutathione peroxidase, and catalase; (iii) enzymes such as serine proteinases, cysteine proteinases, metalloexopeptidases, carboxypeptidases, and aspartic proteases; (iv) proteinase inhibitors of the serpin, cystatin, alpha-2-macroglobulin, trypsin inhibitor-like (TIL), and Kunitz families; (v) tick-specific protein families including lipocalin (histamine-binding proteins), glycine-rich proteins, and the group of secreted conserved proteins [9]. In addition, proteins related to cytoskeletal functions, protein modification machinery, signal transduction, transporters and receptors, metabolism of carbohydrate, energy, nucleotide, amino acids, and lipid were identified (Fig. 3 and Tables 1, 2 and 3).

#### Heme/iron metabolism-related proteins

The most abundant proteins identified in *H. longicornis* saliva are hemelipoproteins, which are associated with heme/iron metabolic processes. This finding is in agreement with previous studies describing hemelipoproteins in proteomic saliva of *Ornithodoros moubata*, *Rhipicephalus microplus*, *Ixodes scapularis* [13, 16, 30, 31], as well as with a study about *A. americanum* immunoproteome [32], which showed that hemelipoproteins are the major proteins in tick saliva.

Hemelipoproteins were first described as heme-binding proteins from tick hemolymph, being able to transport cholesterol, phospholipids, and free fatty acids, in addition to heme [62, 63]. These proteins are the most abundant proteins in spontaneously detached fully engorged *H. longicornis* saliva, which were relatively highly expressed in nymph saliva (Fig. 3 and Table 4). This data is in accordance with the findings observed for *R. microplus*, where hemelipoproteins were highly expressed in partially engorged adult ticks, showing a higher expression in the early developmental stages of tick feeding [16]. The physiological role of hemelipoproteins in blood meal acquisition is not completely understood. Tissue and vessel dilaceration produced by style of tick feeding and the presence of hemoglobin digestive enzymes (such as cathepsin and legumain) in tick saliva cause hemolysis and heme release in the feeding site. It is known that heme activates innate immune cells such as macrophages and neutrophils through activation of innate immune receptors [64–66], thus a role of hemelipoproteins being injected into the feeding site to prevent heme-induced inflammation is suggested. In addition, these proteins could be involved in a heme-excretory system, removing heme excess from tick and re-injecting it into the host. However, the presence of heme in tick saliva needs further investigation.

#### Proteinases

Several proteinases classes were identified in *H. longicornis* saliva: (i) serine proteinases; (ii) cysteine proteinases (including longipain, legumain and cathepsin L); (iii) aspartic proteinases; (iv) serine carboxypeptidases; and (v) proteins that belong to peptidase family M20 from MEROPS (including dipeptidases, and metalloexopeptidases). This set of different proteins present in tick saliva could have multiple modes of action during blood feeding. Serine proteinases may interfere with host inflammation and blood clotting. As shown by the presence of *I. scapularis* saliva protein C activator, a protein acting in the production of activated protein C, a potent anticoagulant that also regulates a myriad of inflammatory responses through protease activated receptors activation [67].

Cysteine proteinases, aspartic proteinases, serine carboxypeptidases and legumains have been described mainly as digestive enzymes with a role in hemoglobin digestion [68, 69] and pathogen transmission in ticks [70]. Thus, the presence of these enzymes in the feeding site may indicate that they act as digestive enzymes secreted into the host, digesting blood components at the tick attachment site and facilitating pathogen transmission during tick feeding. The presence of putative digestive enzymes in tick saliva was also observed in *R. microplus* [16].

In general, dipeptidases hydrolyze the late products of protein degradation to complete the conversion of proteins to free amino acids. In ticks, dipeptidases were reported to be responsible for the destruction of bradykinin, a potent pain inductor [71]. These enzymes are related to the kininase activity found in *I. scapularis* saliva, which may be responsible for the lack of host pain response subsequent to attachment and feeding [72].

#### Proteinase inhibitors

Host defense responses triggered by tick feeding are mainly dependent on the action of several proteinases, such as procoagulant (thrombin, factor Xa and other coagulation factors), pro-inflammatory (neutrophil elastase, proteinase-3, chymase, tryptase, kallikrein, cathepsin L, cathepsin B, cathespin S, cathepsin C, and cathepsin G) and complement enzymes (factors B, C, D, and component 2) [9–12]. Proteinases from these pathways are regulated by specific endogenous inhibitors, maintaining homeostasis. From this perspective, proteinase inhibitors secreted into the feeding site act by disrupting host defenses, facilitating blood meal acquisition. Several families of protease inhibitor domains were found in saliva of *H. longicornis* (Tables 1, 2 and 3).

Serpins are a superfamily of serine proteinase inhibitors involved in blood coagulation, fibrinolysis, inflammation, and complement activation in mammals [73, 74]. These proteins were found mostly in nymph saliva (Table 1). Tick serpins are secreted into the feeding site to disrupt host defenses against tick feeding, including anticoagulant [75–77] and immunomodulatory responses [78–80]. The potential effect of these proteins on host systems is supported by several studies that demonstrate the mortality and reduced feeding efficiency when several tick species were fed on host immunized with tick recombinant serpins [55–59]. Additionally, serpins in *Aedes aegypti* and *Anopheles stephensi* have been shown to play a role in pathogen transmission [81, 82].

Cystatins form a large superfamily of reversible and tight-binding inhibitors that interact with papain-like cysteine proteases and legumains [83]. Tick salivary cystatins have been described as immunosuppressive and anti-inflammatory proteins [84–87]. Moreover, the importance of cystatins in tick physiology was observed in studies that showed that neutralization of cystatin reduces tick feeding ability [52, 85, 88, 89].

Haemalin is a member of Kunitz-type inhibitors identified in *H. longicornis* saliva. This protein has been described as a thrombin inhibitor, delaying bovine plasma clotting time and inhibiting both thrombin-induced fibrinogen clotting and thrombin-induced platelet aggregation. This protein was described as a midgut protein [29], and this is the first time it is described in *H. longicornis* saliva. Taking into account haemalin function, we suggested that this protein acts as an anticoagulant salivary protein during tick feeding.

TIL (trypsin inhibitor-like) proteins have been reported in several tick sialomes [9, 10] and are described as elastase inhibitors, which also have antimicrobial activity [90, 91]. Alpha-2-macroglobulin are a group of proteins that have been found to inhibit several serum proteinases in vertebrates, including thrombin, factor Xa and kallikreins [77, 92–94], mediating T-cell proliferation and activating macrophages [95, 96]. Thus, as these proteins are secreted both in nymph and in adult tick saliva, they could act as anticoagulant and/or immunomodulatory proteins during blood feeding.

#### Tick-specific protein families

Advances in transcriptomic and proteomic studies of tick salivary gland have created new opportunities to identify the variety of tick salivary transcripts and proteins. Many proteins are described to have no similarities to non-tick proteins from the NCBI database [9]. The secreted conserved protein group is composed mainly of tick proteins containing a signal peptide predicted and with similarities to proteins identified in other ticks [9]. Most of proteins included in this group have unknown functions and were described only in gene or protein sequencing projects, having their expression up-regulated after blood acquisition [97, 98]. A functionally described member of tick secreted conserved protein group is *A. americanum* AV422 protein. This protein was first described as an up-regulated protein in response to tick host exposure and/or to feeding stimuli in rabbits [99]. AV422 is secreted into the host during tick feeding, acting as an anticoagulant and anti-complement protein [100, 101]. In *H. longicornis*, this protein is identified in both the spontaneously detached fully engorged nymph and adult saliva (Table 3). Additionally, an AV422-like protein was identified in partially and fully engorged *R. microplus* female saliva proteome [16]. Based on the high amino acid conservation and on its presence in other tick proteomes, it will be interesting to determine whether or not *H. longicornis* AV422-like protein is functionally similar to that of *A. americanum*.

The antigen 5 protein family is a group of cysteine-rich secreted proteins [102]. This group is described in the salivary glands of blood-sucking insects and ticks, with functions that remain mostly unknown [10, 103, 104], being identified exclusively in nymph saliva (Table 1). Glycine-rich proteins are extracellular matrix proteins and/or structural proteins with an important role in attachment to the host, since it they are present in cement material secreted by salivary glands during feeding process [105, 106]. The expression levels of these proteins are associated with size of mouthparts and the number of hosts used by tick during its life cycle. Ticks with short mouthparts and one-host ticks express more glycine-rich protein. It could be implied that one-host ticks are more consistently pressed to sustain attachment on the host’s skin [107]. In this sense, glycine-rich proteins were identified mostly in nymph saliva (Table 1). These proteins are also involved in the defense against pathogens, since they are inducible antibacterial proteins predominantly active by Gram-negative bacteria presents in insect and tick hemolymph and salivary gland [108].

Tick proteins of the lipocalin family are classified based on their homology with tick proteins containing the characteristic tick histamine-binding domain (PF02098) [109, 110]. They are a group of multifunctional secreted proteins that bind several types of small hydrophobic ligands. The role as transport proteins is well studied, however it is clear that the arthropod lipocalin family is involved in various other physiological functions such as cell growth and metabolism, regulation of the immune response, and tissue repair [111]. Lipocalins were detected in several tick saliva proteomes [13, 16], and *H. longicornis* saliva contains at least three proteins described as lipocalin. The presence of lipocalins in tick saliva is related to control of inflammatory processes and interference with host hemostatic functions [112–114]. Furthermore, the importance of lipocalins in nymph and adult saliva has been demonstrated, since the expression of lipocalins in insects and ticks is up-regulated in response to injury [115], as well as to viral [116] or bacterial infections [117], enhancing tick immune responses and resilience to infection.

#### Detoxification/oxidation

In nymph and adult saliva, various proteins involved in processes of detoxification and redox buffering were detected, including glutathione S-transferase, aldehyde dehydrogenase, sulfotransferase, and catalase. These proteins could detoxify oxidants generated during blood meal acquisition and/or host oxidants associated with inflammation. The antioxidant expression levels differ throughout arthropod development [118]. In *H. longicornis* saliva, the abundance of antioxidant proteins is higher in adults, when compared to nymphs (Tables 1, 2 and 3). A glutathione peroxidase was the only protein of this class identified in nymph saliva. This protein has been well characterized for its antioxidant and anti-inflammatory activity in mammals [119], and could be related as an immunomodulatory protein from *H. longicornis* saliva. Sulfotransferase may inactivate dopamine, the secretagogue found in the salivary gland of ticks [120]. Glutathione S-transferase expression has been associated with resistance to acaricides and insecticides in many species [121, 122]. In addition, it has been proposed that GST secreted by parasites has immunomodulatory activity due to the alteration of the cytokine gene expression profile, modulation of immune cell proliferation, and decrease in oxidative ability of phagocytes [123, 124]. The roles of GST and other proteins in the detoxification of endogenous toxin preventing and repairing the damage of ROS generated by hemoglobin degradation has been described [125], and the induction of the expression of these proteins in response to oxidative stress have been observed, supporting the antioxidant physiological role [126, 127]. Furthermore, immunization experiments showed the potential use of tick GST to protect hosts against tick infestation [50, 51].

#### Cytoskeletal

Proteins in *H. longicornis* saliva associated with cytoskeletal and structural cellular function were identified, including actin, tubulin, paramyosin, among others, which are fundamental to intracellular transport and cellular division. Notably, these proteins were abundantly expressed in adult saliva, when compared to nymph saliva, suggesting that the presence of such proteins has a physiological explanation, as opposed to tick/host tissue contamination during saliva collection (Table 4). Since these are intracellular proteins, and considering that most lack signal peptides, it may be hypothesized that they are released as consequence of damage, degeneration, or apoptosis of salivary gland acines [128, 129]. Furthermore, the presence of apocrine secretion in tick salivary gland is described [130]. Another structural protein has been identified in *R. microplus* saliva is paramyosin. This protein is secreted in saliva and recognized during the tick infestation, further suggesting that it may possess additional, non-muscle functions in the tick-host relationship [131]. Troponin I-like molecule was detected in *H. longicornis* saliva, with angiogenesis inhibitor activity impairing host tissue repair and helping the tick feeding process [132]. These observations suggested that these proteins could have roles other than structural functions.

#### Metabolism

A wide variety of enzymes and proteins related to carbohydrate, lipid and amino acid metabolism and to the energetic pathways was observed in *H. longicornis* saliva. This finding is in accordance with other tick saliva proteomes. Similarly to cytoskeletal proteins, proteins that belong to this class are predominant in adult tick saliva (Table 1, 2 and 3). Although functional activity of these proteins remains unknown concerning tick feeding, the activity of some proteins related to metabolism has been characterized.

A salivary enolase from *O. moubata* was described acting as a plasminogen receptor, and may play a role stimulating host fibrinolysis and maintaining blood fluidity during tick feeding [133]. Similar activity was described for enolases from other parasites [134, 135].

Another group of metabolism-related proteins with a possible role in tick feeding is formed by chitinases. Chitinases are either active or inactive, based on the functional domain. Active chitinases are mostly described as responsible for the hydrolytic cleavage of the β-glycosidic linkages between GlcNAc residues of chitin, involved in molting and growth of arthropods [136]. On the other hand, inactive chitinases were suggested to be involved in the maintenance of a stable feeding site and in the activity of a potential immunoglobulin G binding protein in *A. americanum* [137]. It is interesting to note that we show the presence of a putative inactive chitinase only in nymphs (Table 1). However, previous studies showed the presence of an inactive chitinase in adults, as a secreted saliva protein [16, 138].

Enzymes of the o phospholipase A2 family play important roles in phospholipid digestion, rearrangement of cellular membrane phospholipid structures, inflammatory responses, defense and predation mechanisms, and signal transduction [139]. These enzymatic activities have been identified in tick saliva, and are speculated to stimulate tick prostaglandin E2 production [140]. Tick saliva proteomic studies have identified these proteins [16], pointing to their presence in tick saliva. A phospholipase A2 was identified exclusively in nymph saliva (Table 1). These proteins could provide the anti-inflammatory, anti-hemostatic, and vasodilator activity required for long-term blood feeding.

It should be noted that these metabolism-related proteins have been identified in tick saliva, and may be classified as moonlighting proteins, so they can have other distinct functions [141, 142]. Several studies on moonlighting proteins are being carried out, and the discovery of new functions could afford deeper insights into metabolism-related proteins in tick feeding physiology.

### Differential expression between nymph and adult female saliva proteins

Ixodid ticks begin attachment by cutting into the host skin, followed by secretion of cement, a process that may take from one to two days. When completely affixed to the wound site, these ticks feed slowly from the pooled blood formed, for several days [143–145]. During feeding, the salivary gland of adult ixodid ticks undergoes remarkable growth and differentiation, which is accompanied by significant increases in the rate of proteins synthesis [46, 146]. It has been proposed that different tick feeding conditions might affect salivary gland transcription of hard ticks. This feature is related to different vertebrate host exposure and distinct developmental stages, leading to changes in salivary transcription dynamics, as shown previously [14, 16, 32, 99, 147–150].

As shown in other tick saliva proteomes [14, 16], the protein content of tick saliva in different developmental stages varies, such as in this case from nymph to adult female ticks in *H. longicornis*. Of the 135 proteins detected in *H. longicornis* saliva, 30 proteins were identified in nymph saliva, 74 proteins were identified in fully engorged adult females, and 31 were detected in both stages (Fig. 2, Tables 1, 2 and 3). Nymph-specific proteins are represented mostly by proteinase inhibitors and a secreted conserved proteins group (Fig. 3 and Table 1), while adult specific proteins are represented primarily by proteins related to energy metabolism, oxidant/detoxification metabolism, and cytoskeleton (Fig. 3 and Table 2). This finding in adult saliva may be related to salivary gland degeneration starting after adult ticks detach from the host [128, 151].

Changes in expression levels were observed for 11 of the 31 identified tick proteins detected in both stages. The changes in the protein secretion were determined by pairwise comparison between nymphs and fully engorged *H. longicornis* female tick saliva using the TFold module from PatternLab for Proteomics platform [37], and were shown to be statistically significant (Fig. 4 and Table 4). The range in fold change was shown to be greater in the specific-tick proteins than in host protein secreted in nymph saliva. The most significantly affected nymph up-regulated proteins were a hemelipoprotein (up-regulated 34.69-fold), a serpin (up-regulated 8.99-fold), a ribosomal protein s27a (up-regulated 7.98-fold), a cystatin (up-regulated 5.47-fold), and a glutathione peroxidase (up-regulated 5.46-fold). The physiological meaning of these differences is not clear. However, as discussed previously, hemelipoproteins are known by their heme-binding function [63], and expression in early developmental stages of tick feeding observed here is in accordance with that found in *R. microplus* [16]. Proteinase inhibitors were described as early stage secreted saliva proteins in tick, with anticoagulant and immunomodulatory properties, modulating host defense systems trigged against tick feeding [14, 16, 75, 76, 78, 87]. This observation can be related to the nature of the blood feeding process, throughout the different stages of tick as they have to use unique proteins to counteract host defenses, especially for fully engorged nymphs, which need to molt into adults and prepare to feed on another host.

### Host proteins

As shown previously for other tick species [13, 15, 16, 30, 31], a large number of host proteins were identified both in nymph and adult *H. longicornis* saliva (Table 1, 2 and 3). It was demonstrated that ticks transport intact proteins across the digestive system to the hemolymph. After blood ingestion, host blood proteins such as albumin and immunoglobulin cross the midgut epithelium of ticks, and are detected in tick tissues, including secretion into saliva [15, 152, 153]. This finding suggests that the presence of host proteins in tick saliva may be a real and common recycling system present in ticks, not a result of contamination during saliva collection.

In *H. longicornis* saliva, 100 proteins matched the rabbit database. Host identified proteins in tick saliva included proteins related to (i) immunity, such as immunoglobulins, complement system proteins, and antimicrobial proteins; (ii) heme/iron metabolism-related proteins, like serum albumin, peroxiredoxin, serotransferrin, apolipoprotein, and hemopexin; (iii) proteinase inhibitors of the serpin and alpha-2-macroglobulin superfamilies; and (iv) hemoglobin/red blood cells-related products. A set of 13 rabbit proteins was found only in nymph saliva samples, mostly rabbit keratin (Table 4, Fig. 3, and Additional file 3: Figure S1), suggesting that host keratin from tick mouthparts reached saliva during collection, since cleaning of host tissue in nymphs’ mouthparts is more critical than in adults, due to its smaller size. Forty-eight rabbit proteins were only found in this adult saliva, and 30 rabbit proteins were present in both samples (Fig. 2).

The presence of different classes of host proteins in the saliva of the two tick developmental stages suggests the existence of a selective uptake process for host proteins (Fig. 3 and Additional file 3: Figure S1, Tables 1, 2, and 3) as observed in other studies [15, 16]. Furthermore, the relationship between concentrations of these proteins in saliva is different from that observed in host blood. This data is similar to findings observed in other tick species [30, 31, 153, 154]. An explanation for the presence of host proteins in tick saliva is that ticks recycle pivotal host proteins in order to subvert their role in the host and/or using host proteins in specific tick physiologic systems. Utilization of host hemoglobin as substrate to generate antimicrobial peptides against microorganisms was observed in *R. microplus* [155] and *O. moubata* [156]. As observed in *R. microplus*, here we found the same profile for proteins related to heme/iron metabolism. While the major tick heme-binding protein is secreted into saliva from nymphs, its expression decreases in adults. Reduction of heme-binding proteins in adults was accompanied by an increase in the host heme-binding proteins serum albumin, hemopexin, apolipoprotein, and peroxiredoxin (Fig. 3, Table 1, 2 and 3). These observations could suggest that the tick replaces hemelipoproteins by host derived heme-binding proteins, since hemelipoproteins are used for vitellogenesis at the end of the feeding process [62]. The host-derived transferrin was described in the hemolymph of *D. variabilis* and in whole nymphal ticks of *A. americanum* [157, 158]. Recently, a study showed the movement of host-derived transferrin *H. longicornis*, particularly from the midgut to the ovary, via hemolymph [159]. In the same way, *R. microplus* re-use heme from blood meal to synthetize heme proteins during protein synthesis [160].

Mammalian serpins are described endogenous regulators of host defenses against tick feeding [161, 162]. Host proteins of the serpin superfamily were identified in saliva, including alpha-1-antiprotease, antithrombin III, and alpha-2-antiplasmin. These proteins regulate enzymes such as neutrophil elastase, thrombin, and plasmin. It is important to find out whether these host proteins have the potential to inhibit their own serine proteinases. The presence of immunoglobulin chains could be explained as a tick self-defense system, since antibodies remain in an active form in tick hemolymph [152].

These observations suggest that the use of host proteins in tick physiology is not an unusual occurrence, and that these proteins may have an important physiology role in tick feeding process.

## Conclusions

*H. longicornis* tick saliva has not been previously studied due to the considerable difficulty to collect saliva. Previously, researchers used salivary gland extract instead of saliva for protein analysis. This study describes the first proteome analysis of saliva of nymph and adult *H. longicornis*. Despite the use of saliva of fully engorged ticks, we could identify several tick proteins that can provide useful information for basic and applied aspects of the host-parasite interaction. The role of saliva proteins in the contact between a tick and the host is crucial during feeding process, and the knowledge about salivary components may improve the understanding of tick physiology, aiding the identification of a new target for tick control.

## Additional files

Additional file 1: Table S1. Tick proteins in *H. longicornis* saliva identified by mass spectrometry. Name, classification based on function and/or protein family and annotation and accession numbers of best match identities obtained using BLASTP against the several protein databases are provided in the accompanying spreadsheet (Hyperlinked Excel spreadsheet, zipped). Additional file 2: Table S2. Host proteins in *H. longicornis* saliva identified by mass spectrometry. Name, classification based in function and/or protein family and annotation and accession numbers of best match identities obtained using BLASTP against the several protein databases are provided in the accompanying spreadsheet (Hyperlinked Excel spreadsheet, zipped). Additional file 3: Figure S1. Functional classification of *Haemaphysalis longicornis* salivary proteins identified in fully engorged nymph (nymph) and fully engorged adult females (adult). Host proteins detected in nymph (A and C) and adult (B and D). Proteins divided as detected both in nymph (C) and adult (D) or detected exclusively in nymph (A) or adult (B) and classified in groups according to their function and/or protein family. Pie charts represent the percentage of proteins found in each group with respect to normalized spectral counting for each sample.
