# Supplementary figures and images for: Saliva from nymph and adult females of Haemaphysalis longicornis: a proteomic study
Source: Parasit Vectors. 2015 Jun 24;8:338. doi: 10.1186/s13071-015-0918-y (PMC4484640; doi:10.1186/s13071-015-0918-y)

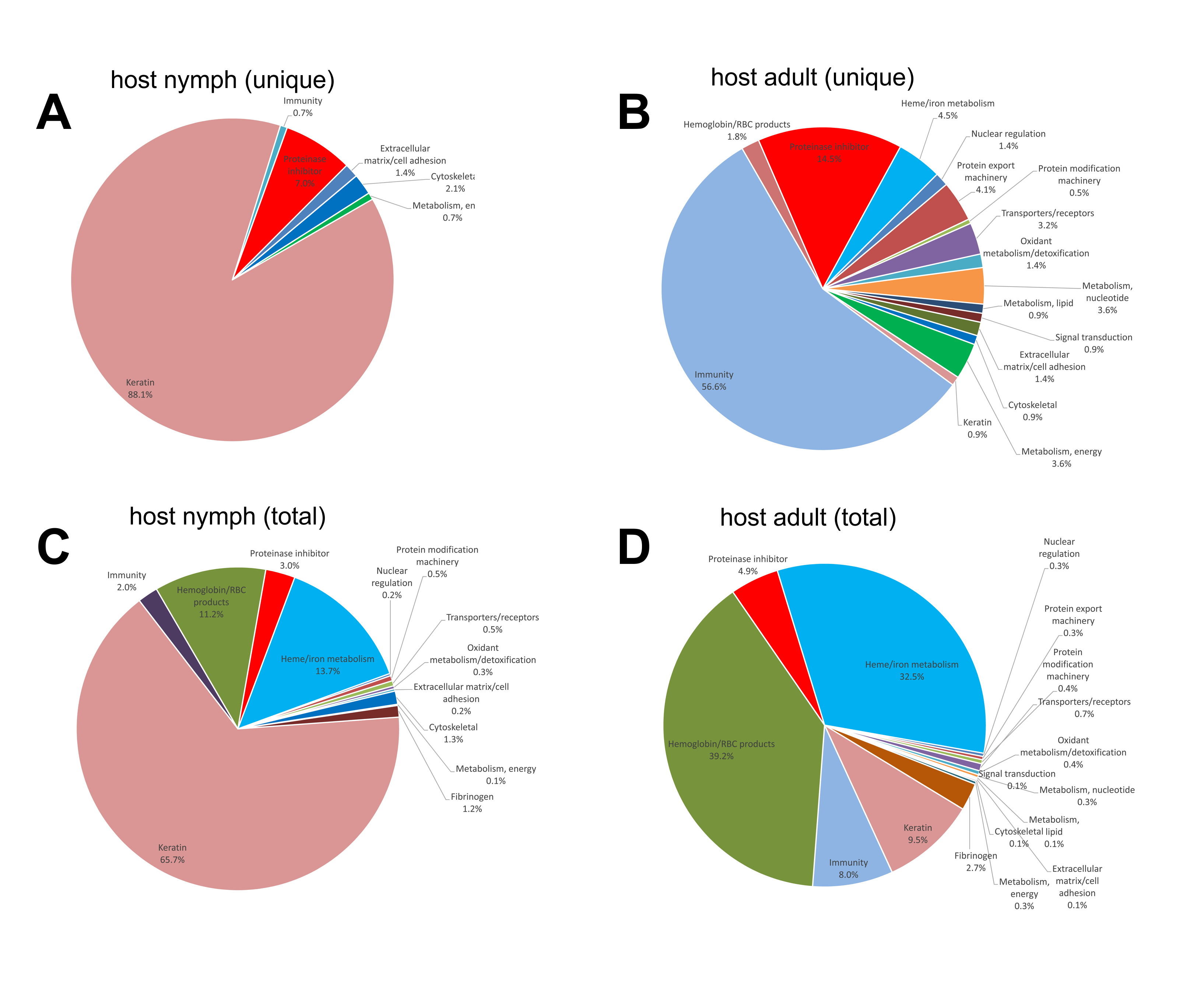

Supplement: Additional file 3: Figure S1. — Functional classification of Haemaphysalis longicornis salivary proteins identified in fully engorged nymph (nymph) and fully engorged adult females (adult). Host proteins detected in nymph (A and C) and adult (B and D). Proteins divided as detected both in nymph (C) and adult (D) or detected exclusively in nymph (A) or adult (B) and classified in groups according to their function and/or protein family. Pie charts represent the percentage of proteins found in each group with respect to normalized spectral counting for each sample. [file 13071_2015_918_MOESM3_ESM.tif]
